# Physiological Responses and Adaptations to Lower Load Resistance Training: Implications for Health and Performance

**DOI:** 10.1186/s40798-023-00578-4

**Published:** 2023-05-12

**Authors:** Jonathon Weakley, Brad J. Schoenfeld, Johanna Ljungberg, Shona L. Halson, Stuart M. Phillips

**Affiliations:** 1 School of Behavioural and Health Sciences, Australian Catholic University, 211.1.26, Brisbane, QLD Australia; 2 Sports Performance, Recovery, Injury and New Technologies (SPRINT) Research Centre, Australian Catholic University, Brisbane, QLD Australia; 3 Carnegie Applied Rugby Research (CARR) Centre, Carnegie School of Sport, Leeds Beckett University, Leeds, UK; 4 Department of Health Sciences, CUNY Lehman College, Bronx, NY USA; 5 The University of Queensland Diamantina Institute, The University of Queensland, Brisbane, QLD Australia; 6 Department of Kinesiology, McMaster University, Hamilton, ON L8S 4K1 Canada

**Keywords:** Muscle mass, Strength, Hypertrophy, Function, Health, Exercise prescription

## Abstract

Resistance training is a method of enhancing strength, gait speed, mobility, and health. However, the external load required to induce these benefits is a contentious issue. A growing body of evidence suggests that when lower load resistance training [i.e., loads < 50% of one-repetition maximum (1RM)] is completed within close proximity to concentric failure, it can serve as an effective alternative to traditional higher load (i.e., loads > 70% of 1RM) training and in many cases can promote similar or even superior physiological adaptations. Such findings are important given that confidence with external loads and access to training facilities and equipment are commonly cited barriers to regular resistance training. Here, we review some of the mechanisms and physiological changes in response to lower load resistance training. We also consider the evidence for applying lower loads for those at risk of cardiovascular and metabolic diseases and those with reduced mobility. Finally, we provide practical recommendations, specifically that to maximize the benefits of lower load resistance training, high levels of effort and training in close proximity to concentric failure are required. Additionally, using lower loads 2–3 times per week with 3–4 sets per exercise, and loads no lower than 30% of 1RM can enhance muscle hypertrophy and strength adaptations. Consequently, implementing lower load resistance training can be a beneficial and viable resistance training method for a wide range of fitness- and health-related goals.

## Key Points

- Lower load (i.e., < 50% of 1RM) resistance training can be a viable and effective method of developing muscle hypertrophy and strength. Furthermore, it can have tangible benefits for healthy populations and those at risk for developing chronic diseases.
- Despite hesitancy and skepticism over the practicality of lifting with lower loads for muscle hypertrophy and strength, there is substantial evidence that supports its implementation.
- Considering the somewhat discordant cellular signaling differences between lifting with lower and higher loads, the practical significance of these findings still need to be elucidated.
- To maximize the benefits of lower load resistance training, high levels of effort and training in close proximity to concentric failure are required. Furthermore, it could be recommended that it is implemented 2–3 times per week with 3–4 sets per exercise, and loads no lower than 30% of 1RM.
- Lower load resistance training can be used in conjunction with higher loads (i.e., < 50% of 1RM) and should be a personal choice based on individual goals. This may help reduce participation barriers and promote exercise adherence.

## Background

Resistance training is an important consideration for health and performance. The physiological responses and adaptations induced by resistance training are infinitely variable and are determined by acute training variables (e.g., intensity, volume, and muscle action). The American College of Sports Medicine (ACSM) recommends it be performed with a load of at least 70% of one-repetition maximum (1RM) when aiming to induce muscle hypertrophy [1]. Furthermore, loads ≥ 80% are commonly recommended for strength development in trained individuals [1]. However, despite firmly held beliefs within the exercise world, when standardized through work-matched or training to momentary concentric failure, lower load resistance training (i.e., loads < 50% 1RM) can serve as an effective alternative to traditional higher load (i.e., loads ≥ 70% 1RM) training and can induce similar or even superior changes in a wide range of physiological, performance, and health-related outcomes [2–5].

Physical inactivity is a leading cause of death worldwide and has substantial economic, environmental, and social consequences [6, 7]. Considering the benefits of resistance training, it is now commonly recommended in health guidelines [8, 9], but despite its importance, participation in resistance training is relatively low. For example, in Australia, only 10.4% of adults meet resistance training recommendations [10]. Consequently, promoting a range of methods that are practical, accessible, and encourage adherence may be beneficial for health outcomes. The use of lower load resistance training may be one of these methods, as it can stimulate adaptations comparable to higher load resistance training [2, 4]. In addition, lower-load training may reduce articular stress compared to higher-load training; which could be of particular importance for those with joint-related issues (e.g., osteoarthritis). Moreover, lower loads may allow for the completion of resistance training without the need for specific facility memberships and can enable the maintenance or augmentation of physical qualities during periods where higher load training is not feasible. This may be particularly pertinent in the current climate, considering that a pandemic has forced periods of isolation and reduced access to resistance training equipment. Here, we review the mechanisms responsible for improvements in skeletal muscle and physical performance with specific emphasis on lower load resistance training. Additionally, we discuss some of the potential implications and applications for health-related outcomes and physical performance in healthy populations and those at risk.

## Main Text

### Physiological Responses Following Lower Load Resistance Training

A commonly investigated method of applying lower load resistance training has been to use loads of 30% 1RM and with repetitions taken to volitional or concentric failure [2–4, 11–15]. Participants have typically performed three to four sets across 8–12 week periods; however, substantial changes in strength and leg fat-free mass and skeletal mass have been observed in as little as two weeks [3]. When chronic interventions have compared traditional higher load resistance training (i.e., 80–90% 1RM) to lower load (i.e., ~ 30% 1RM) training, similar training-induced improvements in body composition and some measures of neuromuscular performance have occurred, despite the significant differences in external load lifted [4, 5]. In addition to similarities in changes in fat-free mass and non-specific measures of strength, research indicates comparable improvements in type I and type II cross-sectional area (CSA) [5, 13], pennation angle [15], rate of force development [4], and satellite cell activity between lower and higher load conditions [13]. Furthermore, lower load resistance training is accompanied by greater increases in specific mitochondrial proteins (i.e., mitochondrial fission 1 protein, dynamin-related protein, optic atrophy type 1, and parkin) [13].

Given the ability of lower load resistance training to promote increases in muscle mass, a range of molecular mechanisms that underpin skeletal muscle adaptations have been investigated [4, 5, 13]. Evidence suggests that when exercise is completed with a controlled tempo of one second eccentric and concentric durations (i.e., 1/0/1/0), exercise effort is an important factor influencing the early amplitude of myofibrillar protein synthesis [2]. Additionally, the duration of the myofibrillar response at extended time points (e.g., 24–72 h) may be determined by the exercise volume completed rather than the load lifted [2, 3, 11], with greater responses in trained individuals when 30%, rather than 90%, of 1RM loads, were taken to the point of concentric failure [11]. It should also be noted that lower loads with higher absolute volumes have been shown to cause sustained sarcoplasmic protein synthesis 24 h post-exercise [11]. This finding indicates that training with lower loads can increase proteins from all fractions in muscle [13] and may lead to enhanced oxidative capacity and hypertrophy [11, 16].

**Fig. 1:**
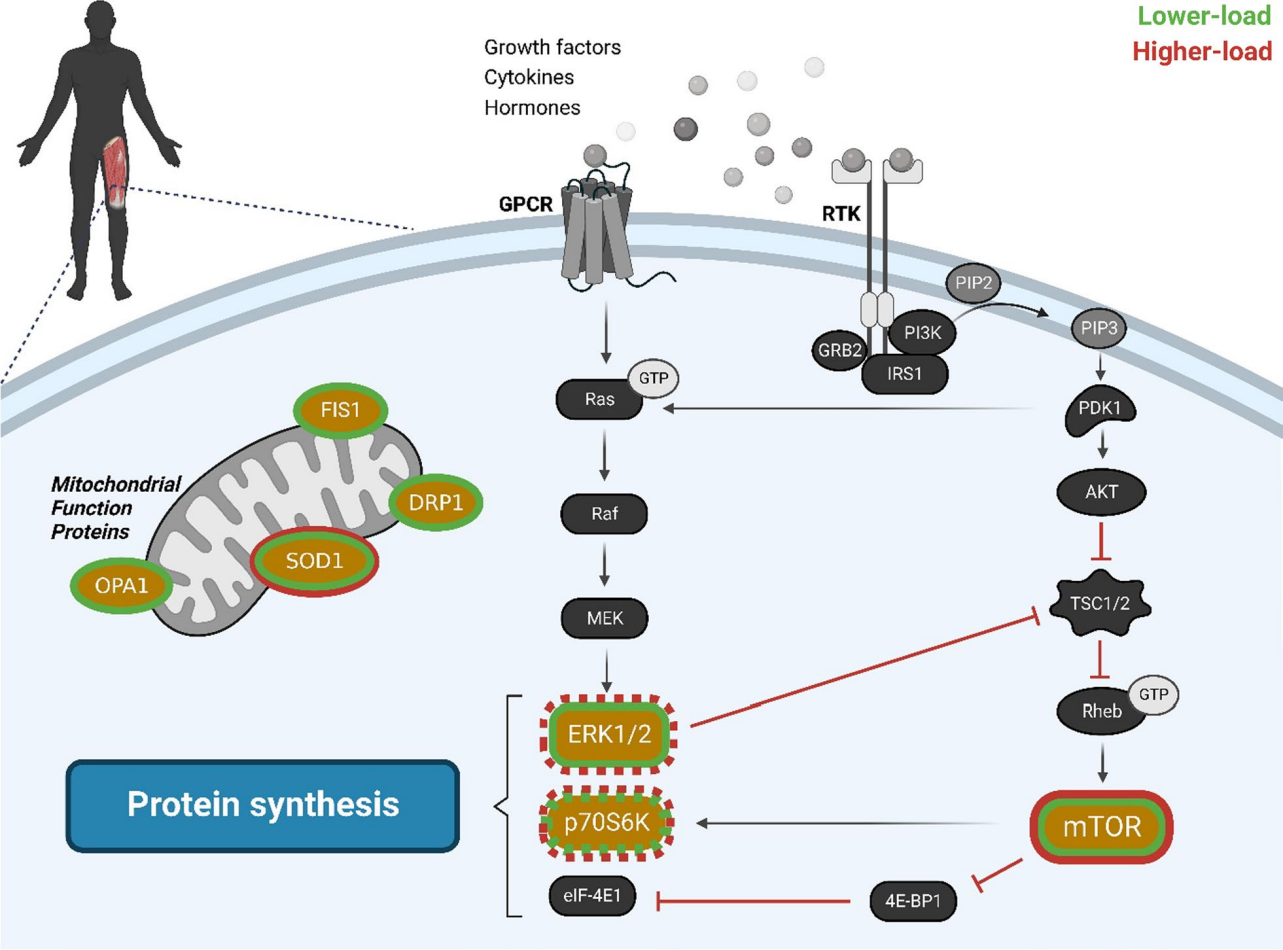
Acute (1 and 4 h) changes in molecular signaling pathways following either lower (30–40% of 1RM) or higher load (80–90% of 1RM) resistance training. Dashed colored outline indicates increased expression at either 1 or 4 h timepoints. Full outline indicates increased expression at both 1 and 4 h timepoints. Additionally, increases in the chronic expression of mitochondrial function proteins are presented. Data are from [4, 5, 13, 15]

Several studies have investigated the upstream signals that initiate changes in muscle protein synthesis in response to various loading schemes, with evidence of differential changes in key signaling pathways (Fig. 1) [2, 11, 13]. In trained and untrained participants, increased phosphorylation of the mammalian target of rapamycin (mTOR) and its downstream targets have been observed with heavier (80–90% of 1RM) and lighter loads (i.e., 30% of 1RM) at early time points following exercise [4, 11]. Additionally, while significant increases in p70S6K phosphorylation were not observed when training to momentary concentric failure with 30% of 1RM in untrained and recreationally trained participants one hour following exercise, there is only equivocal evidence when using loads of 80% 1RM [4, 17]. However, lower load protocols in trained participants have shown increased phosphorylation responses four hours after exercise [11]. These results suggest that heavier and lighter relative loads lifted until the point of volitional failure may result in a different time course of anabolic signaling, with p70S6K phosphorylation occurring later after exercise with lighter compared with heavier relative loads [4]. Furthermore, while increases in extracellular signal-regulated protein kinases 1 and 2 (ERK1/2) have been observed one hour following exercise with higher and lower loads when trained to volitional failure [17], only lower loads display increases four hours post-exercise [11]. Indeed, previous work suggests that ERK1/2 is sensitive to the volume of contractions during resistance training [18], with this supported by Burd et al. [11], who used a substantially greater number of repetitions (~ 94 vs. ~ 19 repetitions) in their 30% of 1RM to failure condition. Notwithstanding the somewhat discordant patterns of mTOR pathway signaling, muscle protein synthesis rates are similar following both lower and higher load contractions when performed to volitional failure [11]. Thus, the practical significance of these findings remain undetermined.

Underpinning the physiological responses to higher and lower load resistance training are potential differences in motor unit recruitment patterns. Traditional recommendations of higher load resistance training for strength and hypertrophy development are predicated on Henneman’s size principle, which has been extrapolated to imply that lower loads do not provide a sufficient stimulus to innervate the highest threshold motor units associated with type II myofibers [19]. However, the onset of fatigue necessitates the activation of larger (i.e., higher threshold) motor units and thus the full spectrum of fiber types are ultimately recruited when training is carried out with very high levels of effort [17]. While greater surface electromyograph (EMG) amplitudes have been reported with higher vs lower load resistance training, the relationship between EMG amplitude and motor unit recruitment is not easily determined during sustained/fatiguing contractions and can be misleading [17, 20]. Subsequently, using this measure to infer fiber-type-specific motor unit recruitment and longitudinal hypertrophy should be cautiously interpreted [21]. An alternative method of assessing muscle fiber activation is via fiber-type-specific glycogen depletion [17]. This method has been used to demonstrate that when completing resistance training to concentric failure, recruitment, substrate depletion, and phosphorylation of anabolic signaling proteins within type I and II muscle fibers occurs irrespective of load, duration, or volume [17]. Furthermore, these changes in glycogen content have been related to changes in muscle signaling proteins (e.g., phosphorylation of p70S6K), which have established correlations with muscle protein synthesis [11] and changes in CSA following resistance exercise [4].

### Lower Load Resistance Training and Muscle Hypertrophy and Strength Adaptations

Resistance training is an important consideration for any exercise program that focuses on developing strength and improving physical performance. Traditionally, higher-loads have been promoted to elicit gains in lean body mass and strength. Indeed, the National Strength and Conditioning Association (NSCA) has stated that hypertrophy is most efficacious between 7 and 12 repetitions [22]. However, when lower loads (e.g., 30% of 1RM) are taken to volitional or momentary concentric failure, similar improvements in muscle mass can occur when compared to intensities that would enable repetitions within this 7–12 repetition range (i.e., ~ 70–83% of 1RM) [4, 5, 13, 14]. Furthermore, when the total volume of work (load • repetitions • sets • workout frequency) is equated, meta-analytic estimates show that load has no bearing on hypertrophic adaptations [23]. Additionally, lower loads can cause substantial improvements in exercise-specific and non-specific strength [4, 5, 16, 24]. Therefore, lower loads should be considered a viable alternative to higher load training and may augment physical performance from resistance training programs.

Numerous studies have compared the effects of lower and higher load training on muscular adaptations, with the majority demonstrating that lower load training induces comparable increases in muscle hypertrophy compared to higher loads [23, 25]. One important caveat to this, however, is that a relatively high level of effort must be reached to induce these adaptations when using lower loads. Lasevicius and colleagues [14] compared training at 30% or 80% of 1RM with exercise being either taken to 60% of concentric failure or absolute concentric failure in a volume-matched design. While significant improvements in CSA were seen in the 80% conditions and the 30% taken to absolute concentric failure condition, training with 30% of 1RM without concentric failure, despite the equivalent volume of work, did not induce significant changes after eight weeks of training. In contrast to these findings, Nobrega et al. [15] showed that by allowing participants to finish a set at a point of volitional failure (i.e., when the individual voluntarily interrupts the exercise), similar changes in muscle CSA occurred compared to training to momentary concentric failure (i.e., when the concentric action can no longer be completed). As findings from this study showed similar absolute volumes between conditions, it can be inferred that proximity to concentric failure is an essential consideration when aiming to maximize hypertrophic responses and supports recent meta-analytic findings [23].

Despite going against conventional wisdom, lower loads can elicit substantial improvements in strength measures. Load is often emphasized as fundamental for strength development [22], but lower loads can improve indices of strength and may be a useful tool when the handling of higher loads is infeasible or not preferred. While there are inconsistencies as to whether lower loads can induce comparable improvements in absolute strength compared to higher loads, the answer is likely nuanced. When direct comparisons have been made, evidence suggests that lower loads do [4, 5, 15] and do not [14, 16, 24, 26] induce similar strength adaptations. However, subtle differences between studies may help explain discrepancies in conclusions. For example, in studies that have tested maximal dynamic strength of the exercise used throughout the training protocol, the studies that have provided participants with familiarization or an occasional maximal stimulus report similar strength adaptations [4, 5, 15]. Thus, so long as there is the periodic implementation of heavier load training, comparable changes in strength may occur. Furthermore, when the strength assessment is not related to the exercise (e.g., testing isometric strength) and training is taken to concentric failure, strength development is again similar between lower and higher loads [4, 5]. This finding is in-line with previous work showing that strength gains are specific to the trained movement [27] and hence, would favor training at higher loads as those persons would be lifting loads closer to their 1RM and thus getting more practice. Finally, the development of muscular endurance has been shown to be substantially greater when lower loads are used [16]. Although, it should be acknowledged that these results may have been biased by the smaller absolute improvements in 1RM strength attained in the lower load group, which subsequently influenced the load during the post-intervention muscular endurance test. Thus, changes in muscular endurance may be nuanced and are likely influenced by the muscle groups used and the testing methodology implemented [28]. Nevertheless, while speculative, the greater time under tension that lower load training requires has been shown to alter mitochondrial protein synthesis and may improve muscle fatigue resistance and enhancing cellular energetics [2, 13].

Several mechanisms that underpin the strength and hypertrophic adaptations to lower load training have been proposed, with varying evidence supporting their influence. Compared to higher load training, alterations in fiber type [4, 5, 26], neural adaptations [12, 15], and mitochondrial protein synthesis and metabolism [13] have all been suggested to occur with limited to equivocal evidence. Several studies have investigated the influence of lower load, higher volume resistance training on fiber type hypertrophy with conflicting findings. While research [29, 30] has suggested that the greater metabolic stress associated with lower load training may induce greater type I fiber hypertrophy, research by Morton et al. [5] and Mitchell et al. [4] in trained and untrained participants, respectively, showed no differences when higher or lower loads are completed to concentric failure. Moreover, research indicates similar hypertrophy between lower and higher load training in the soleus (a type I dominant muscle) and the gastrocnemii (a mixed fiber muscle) [31]. Holm et al. [26] demonstrated no differences in type I or type IIa fiber adaptations between loading methods, although lifting higher loads induced a greater reduction in type IIX fibers. Changes in muscle pennation angle were investigated by Nobrega et al. [14], with a ~ 9% change when training with lower and higher load conditions, which may help explain similarities in changes in muscle strength. However, following six weeks of training with higher loads may allow for greater neural adaptations [12]; specifically, increased voluntary activation and normalized EMG amplitude during submaximal and maximal torque production [12]. Finally, evidence from a single study [13] has suggested that completing the lower load, higher volume training (compared to higher load, lower volume training) three times per week across 10 weeks can be a more potent stimulus for mitochondrial metabolism and turnover, which may be related to the greater substrate use and metabolic demand induced with lower load training. Such responses may underpin these changes in mitochondrial proteins; however, further research is warranted.

#### Potential Applications of Lower Load Resistance Training in People Who are at Risk

While lower load resistance exercise has been discussed as a method to augment human strength and lean body mass, it should be noted that these outcomes are essential components of healthy living. Low muscle mass and strength are associated with poor physical function and are associated with future mobility impairment in older adults [32, 33]. Additionally, a range of diseases can be positively influenced by enhancing an individual’s strength and lean mass [34]. Consequently, due to the low cost and simple implementation of lower load resistance training, evidence suggests it can be a potent means to reduce chronic disease risk and improve long-term health [34].

Aging is a significant predictor of mobility impairment, with this reduced mobility exacerbating chronic disease [34, 35]. Numerous reviews demonstrate that resistance exercise in pre-frail and frail older adults can significantly enhance muscular strength, gait speed, and physical performance [36, 37]. While higher load resistance exercise (i.e., ≥ 70% of 1RM) has been suggested to be more effective than lower load in mitigating the impairment of mobility [38], heterogeneity between studies complicates their comparison and makes it difficult to determine whether one loading condition is superior to another. It should be noted that resistance training, even using a person’s body mass as resistance, can substantially improve physical function, and this can be as effective as traditional resistance training methods that require external loads [39]. In periods of low activity (e.g., immobilization of a single limb and rehabilitation), resistance training can offset anabolic resistance and muscle atrophy [3]; with as little as two weeks of lower load, higher volume resistance training resulted in substantial increases in muscle mass in older adults that completed a period of step-reduction [3]. Finally, lower load resistance exercise may be a practical option for those with reduced mobility. A substantial hurdle to resistance exercise is access to an appropriate facility. Thus, using lower loads (e.g., body mass) that are often more accessible, and completing repetitions to volitional failure or close to concentric failure [15], may be an effective method for improving indices of health.

Lean body mass is essential for the maintenance of metabolic health. Approximately 80% of glucose is deposited in skeletal muscle during postprandial periods [40], and adequate lean mass is important for mitigating insulin resistance and type 2 diabetes in adults. While changes in skeletal muscle mass may alter glucose handling, resistance training and muscle contractions, in general, can improve glucose homeostasis through insulin-dependent and independent signaling pathways [41]. However, the optimal resistance training intensity for metabolic health is unclear, with a review by Gordon et al., [42] noting that higher-load resistance training results in the greatest improvements in glycemic control. Although, this conclusion failed to take into account the total volume of exercise performed. More recent evidence has shown that when matched for exercise volume, there was no significant difference in glycemic control between higher or lower load resistance training (i.e., 70% vs. 50% 1RM) in individuals with type 2 diabetes [43]. While further work is still required, this provides the rationale that simply performing resistance training with sufficient volume, rather than emphasizing the total load, is the more important consideration for glycemic control and metabolic health.

Despite resistance training not having always been endorsed as a mode of exercise for reducing the risk of cardiovascular disease [44], its benefits for this purpose are clear. While it has been suggested that cardiac hypertrophy and subsequent greater risk of mortality may occur when higher pressures are placed on the heart [45], the excessive elevation of blood pressure is only observed with higher loads (i.e., > 70% of 1RM) and is less of a concern when lower loads are implemented [46]. Although, it should be noted that this has not been investigated when higher and lower loads have both been taken to failure. Furthermore, in older adults with cardiovascular disease, low to moderate load resistance training (i.e., 30–69% of 1RM) has been associated with lower rates of adverse cardiovascular complications than aerobic exercise [47]. With strength and skeletal muscle independently associated with risk for cardiovascular disease and mortality [48–50], resistance training has been posited as an important interventional strategy for mitigating cardiovascular risk [34]. Finally, as low and moderate loads have been demonstrated to exert similar improvements in a host of cardiovascular risk factors (e.g., blood pressure [51] and blood lipid profiles [52, 53]), the use of relatively light external loads could support the health and well-being of individuals at risk of, or currently suffering from, cardiovascular disease.

#### Application of Lower Load Training for Physical Development and Health

With the progression of modern society, improvements in technology, and continued decreases in physical exercise, there is perhaps no time in history where completing dedicated resistance training has been more important to public health. Ironically, implementing traditional higher-load resistance training may be difficult in the current climate considering the pandemic, periods of enforced isolation, and reduced access to dedicated training equipment. Therefore, lower load resistance exercise may act as an increasingly important method in helping improve health and physical performance. Indeed, considering the common factors that hinder the implementation of traditional higher load training (e.g., access, equipment, and apprehension of heavy loads), one of the many benefits of this form of training is that it requires minimal equipment. Thus, lower load resistance training may substantially benefit those who need to offset the loss of muscle mass and strength during periods where access to traditional forms of resistance training is limited (e.g., travelling and quarantine). Alternatively, the benefits of inducing substantial amounts of muscle hypertrophy and strength may extend to those with limited mobility or those rehabilitating from injury [3].

To maximize the benefits of lower load training, substantial effort is required, which can lead to high levels of discomfort [54]. It is important to make individuals aware of this outcome and differentiate between effort and discomfort, and discrepancies in the literature may be attributed to these factors. It has been posited that individuals may find it more difficult to reach momentary concentric failure with lower loads due to greater levels of discomfort [55]. However, to recruit motor neurons that innervate type II fibers using lower loads, there is a need to take training close to, if not to, concentric failure [17]. Evidence suggests that with lower loads, even when training is volume-matched between concentric failure and non-failure conditions, proximity to repetition failure is needed to maximize physical development [14]. It should be acknowledged, however, that strength improvements can still be achieved with lower loads even when sets are finished at 60% of the volume required to induce concentric failure, and these improvements may be valuable to those not solely focused on maximizing 1RM strength [14].

It should be noted that the use of lower loads does not exclude the use of higher loads. From a practical standpoint, it may be strategic to selectively use lower loads with isolation/auxiliary exercises (i.e., exercises that only use one joint for movement), although multi-joint/compound exercises can also be used. Isolation/auxiliary exercises often have less complexity, enable greater focus on exercising closer to failure, have a lower burden on other muscle groups, and reduce the need for a spotter. Alternatively, higher relative loads may be preferential for improving strength or to mitigate fatigue or feelings of discomfort [56].

**Fig. 2:**
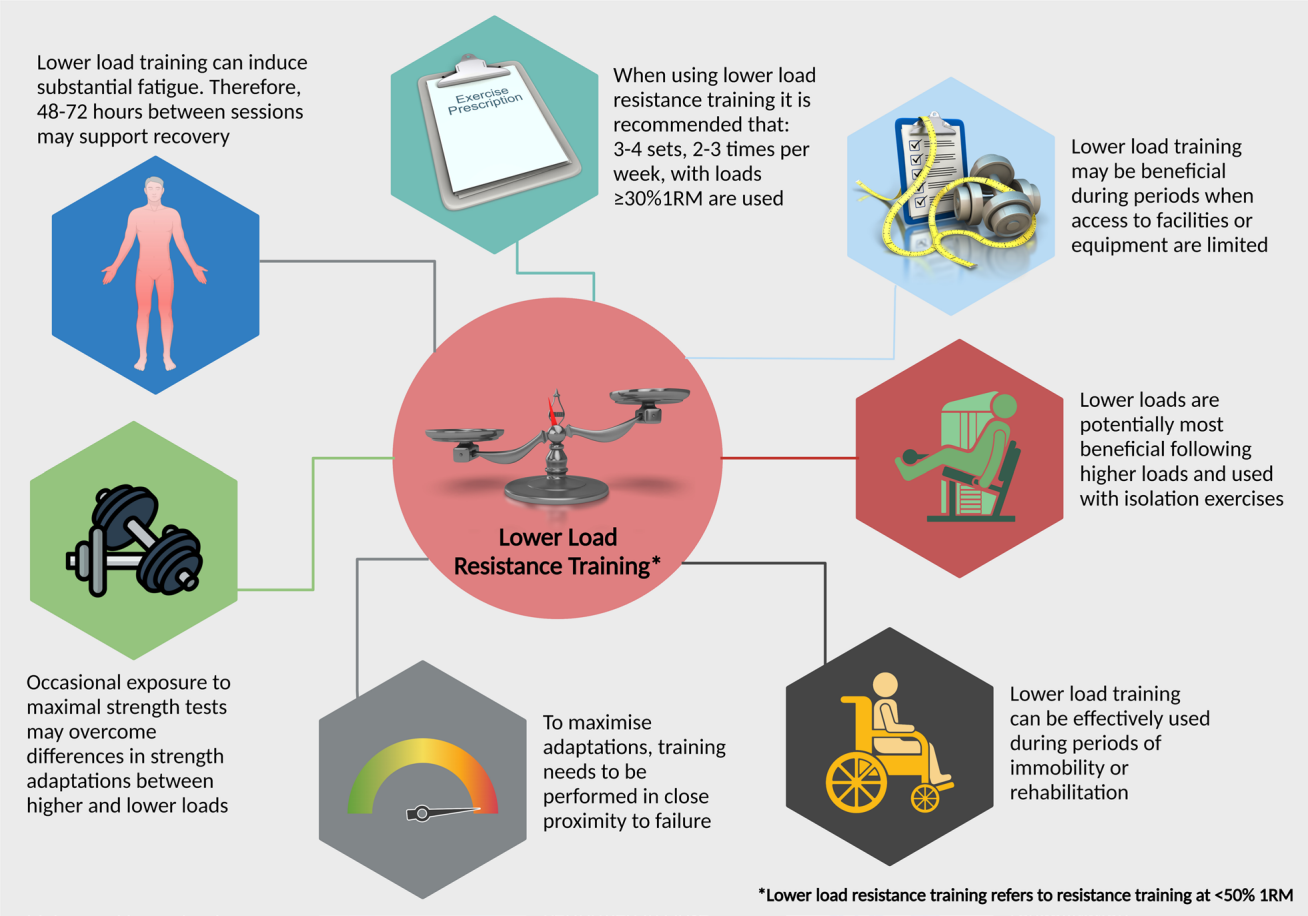
Recommendations and considerations for the application of lower load resistance training

Any training method should be considered within the holistic exercise program, and the implementation of lower load resistance training is no different. As this training method often requires higher volumes of exercise, how it fits within a periodized exercise routine should be carefully evaluated. Furthermore, completing greater volumes of work and training close to concentric failure can cause considerable discomfort [14] and increase recovery time [56, 57]. Therefore, as long as participants understand the need to be within proximity to failure, using volitional interruption (i.e., when the proximity to failure is close and individuals feel sufficient fatigue has been induced) may be an option [15]. Evidence suggests that full motor unit activation can be achieved within 3–5 reps of concentric failure [57], with lower load volitional interruption allowing comparable increases in strength and CSA compared to training with higher loads or lower loads to failure [4, 13, 15, 17]. The use of repetitions-in-reserve and systematic changes in proximity to concentric failure (e.g., week one: three repetitions from concentric failure; week two: two repetitions from concentric failure) could be used. Although, when using repetitions-in-reserve, it is recommended that sets be terminated in close proximity to failure, as this can improve the accuracy of estimation [58]. Furthermore, due to the substantial neuromuscular fatigue induced from lower load training [59], separating exercise sessions by at least 48–72 would seem to be warranted. Finally, research demonstrating substantial and comparable gains in muscle mass and strength with lower loads have completed exercise 2–3 times per week with 3–4 sets per exercise [4, 5, 13–15, 24], and loads no lower than 30% of 1RM [24]. Recommendations and considerations can be found in Fig. 2.

## Conclusions and Future Directions

A substantial body of evidence supports the use of lower load resistance training for inducing improvements in muscle hypertrophy and strength. These improvements have tangible benefits for healthy populations and those at risk for developing chronic diseases. However, despite the evidence available, there is still hesitancy and skepticism over the practicality of lifting with lower loads. We speculate that this hesitancy likely stems from beliefs that heavy loads are necessary for improvements in strength and muscle growth. While evidence of its benefits is compelling, it should be acknowledged that further research is still required to elucidate optimal implementation of lower loads in exercise program design. There is likely a relative intensity threshold with which adaptations are suboptimal, perhaps occurring when loads approximate 20% 1RM [24]. Furthermore, like most forms of resistance training prescription, evidence is needed to understand whether chronic exposure results in differential adaptations. The chronic adaptation to lower load training may be particularly interesting at the fiber level, with evidence suggesting that acute differential signaling and protein synthesis responses may occur, but longitudinal data are currently equivocal. Finally, further investigation is needed to understand the proximity to failure that one must practice to induce adaptations in muscle hypertrophy that are equivalent to higher loads. This knowledge may help reduce the discomfort and fatigue associated with lower load training [56] and improve exercise adherence. Information from these future studies would undoubtedly aid the implementation of this form of training and guide decisions around its use. Furthermore, it may promote accessibility to resistance training and its benefits for health.

## Data Availability

All data and material reported in this review are from peer-reviewed publications.

## Abbreviations

1RM: One repetition maximum
ACSM: American College of Sports Medicine
CSA: Cross-sectional area
mTOR: Mammalian target of rapamycin
ERK1/2: Extracellular signal-regulated protein kinases 1 and 2
EMG: Electromyograph
NSCA: National Strength and Conditioning Association

## References

[1] Ratamess N, Alvar BA, Evetouch T, Housh TJ, Kibler WB, Kraemer WJ (2009). Progression models in resistance training for healthy adults. Med Sci Sport Exerc.

[2] Burd NA, Andrews RJ, West DW, Little JP, Cochran AJ, Hector AJ (2012). Muscle time under tension during resistance exercise stimulates differential muscle protein sub-fractional synthetic responses in men. J Phys.

[3] Devries MC, Breen L, Von Allmen M, Macdonald MJ, Moore DR, Offord EA (2015). Low-load resistance training during step-reduction attenuates declines in muscle mass and strength and enhances anabolic sensitivity in older men. Phys Rep.

[4] Mitchell CJ, Churchward-Venne TA, West DW, Burd NA, Breen L, Baker SK (2012). Resistance exercise load does not determine training-mediated hypertrophic gains in young men. J App Physiol.

[5] Morton RW, Oikawa SY, Wavell CG, Mazara N, Mcglory C, Quadrilatero J (2016). Neither load nor systemic hormones determine resistance training-mediated hypertrophy or strength gains in resistance-trained young men. J Appl Physiol.

[6] Kohl HW, Craig CL, Lambert EV, Inoue S, Alkandari JR, Leetongin G (2012). The pandemic of physical inactivity: global action for public health. The Lancet.

[7] Rhodes RE, Lubans DR, Karunamuni N, Kennedy S, Plotnikoff R (2017). Factors associated with participation in resistance training: a systematic review. Br J Sports Med.

[8] Health DO (2014). Australia's physical activity and sedentary behaviour guidelines for adults (18–64 years).

[9] Organization WH (2012). Recommended levels of physical activity for adults aged 18–64 years.

[10] Bennie JA, Pedisic Z, Van Uffelen JGZ, Charity MJ, Harvey JT, Banting LK (2016). Pumping iron in australia: prevalence, trends and sociodemographic correlates of muscle strengthening activity participation from a national sample of 195,926 adults. PLoS ONE.

[11] Burd NA, West DW, Staples AW, Atherton PJ, Baker JM, Moore DR (2010). Low-load high volume resistance exercise stimulates muscle protein synthesis more than high-load low volume resistance exercise in young men. PLoS ONE.

[12] Jenkins NDM, Miramonti AA, Hill EC, Smith CM, Cochrane-Snyman KC, Housh TJ (2017). Greater neural adaptations following high- vs. low-load resistance training. Front Physiol.

[13] Lim C, Kim HJ, Morton RW, Harris R, Phillips SM, Jeong TS (2019). Resistance exercise–induced changes in muscle phenotype are load dependent. Med Sci Sport Exerc.

[14] Lasevicius T, Schoenfeld BJ, Silva-Batista C, Barros TS, Aihara AY, Brendon H (2019). Muscle failure promotes greater muscle hypertrophy in low-load but not in high-load resistance training. J Strength Cond Res.

[15] Nóbrega SR, Ugrinowitsch C, Pintanel L, Barcelos C, Libardi CA (2018). Effect of resistance training to muscle failure vs. volitional interruption at high-and low-intensities on muscle mass and strength. J Strength Cond Res.

[16] Schoenfeld BJ, Peterson MD, Ogborn D, Contreras B, Sonmez GT (2015). Effects of low-vs. high-load resistance training on muscle strength and hypertrophy in well-trained men. J Strength Cond Res.

[17] Morton RW, Sonne MW, Farias Zuniga A, Mohammad IYZ, Jones A, Mcglory C (2019). Muscle fibre activation is unaffected by load and repetition duration when resistance exercise is performed to task failure. J Physiol.

[18] Williamson D, Gallagher P, Harber M, Hollon C, Trappe S (2003). Mitogen-activated protein kinase (mapk) pathway activation: effects of age and acute exercise on human skeletal muscle. J Physiol.

[19] Duchateau J, Semmler JG, Enoka RM (2006). Training adaptations in the behavior of human motor units. J Appl Physiol.

[20] Vigotsky AD, Ogborn D, Phillips SM (2016). Motor unit recruitment cannot be inferred from surface emg amplitude and basic reporting standards must be adhered to. Eur J Appl Physiol.

[21] Vigotsky AD, Halperin I, Trajano GS, Vieira TM (2022). Longing for a longitudinal proxy: acutely measured surface emg amplitude is not a validated predictor of muscle hypertrophy. Sports Med.

[22] Sheppard JM, Triplett NT. Program design for resistance training. In: Haff GG, Triplett NT, editors. Essentials of strength training and conditioning. Champaign: Human Kinetics; 2015. p. 457.

[23] Carvalho L, Junior RM, Barreira J, Schoenfeld BJ, Orazem J, Barroso R (2022). Muscle hypertrophy and strength gains after resistance training with different volume-matched loads: a systematic review and meta-analysis. Appl Physiol Nutr Metab.

[24] Lasevicius T, Ugrinowitsch C, Schoenfeld BJ, Roschel H, Tavares LD, De Souza EO (2018). Effects of different intensities of resistance training with equated volume load on muscle strength and hypertrophy. Eur J Sport Sci.

[25] Schoenfeld BJ, Grgic J, Ogborn D, Krieger JW (2017). Strength and hypertrophy adaptations between low- vs High-load resistance training: a systematic review and meta-analysis. J Strength Cond Res.

[26] Holm L, Reitelseder S, Pedersen TG, Doessing S, Petersen SG, Flyvbjerg A (2008). Changes in muscle size and mhc composition in response to resistance exercise with heavy and light loading intensity. J Appl Physiol.

[27] Baker D, Wilson G, Carlyon B (1994). Generality versus specificity: a comparison of dynamic and isometric measures of strength and speed-strength. Eur J Appl Physiol Occup Physiol.

[28] Schoenfeld BJ, Grgic J, Van Every DW, Plotkin DL (2021). Loading recommendations for muscle strength, hypertrophy, and local endurance: a re-examination of the repetition continuum. Sports.

[29] Netreba A, Popov D, Bravyy Y, Lyubaeva E, Terada M, Ohira T (2013). Responses of knee extensor muscles to leg press training of various types in human. Ross Fiziol Zh Im I M Sechenova.

[30] Vinogradova OL, Popov DV, Netreba AI, Tsvirkun DV, Kurochkina NS, Bachinin AV (2013). Optimization of training: development of a new partial load mode of strength training. Fiziol Cheloveka.

[31] Schoenfeld BJ, Vigotsky AD, Grgic J, Haun C, Contreras B, Delcastillo K (2020). Do the anatomical and physiological properties of a muscle determine its adaptive response to different loading protocols?. Physiol Rep.

[32] Visser M, Goodpaster BH, Kritchevsky SB, Newman AB, Nevitt M, Rubin SM (2005). Muscle mass, muscle strength, and muscle fat infiltration as predictors of incident mobility limitations in well-functioning older persons. J Gerontol A Biol Sci Med Sci.

[33] Visser M, Kritchevsky SB, Goodpaster BH, Newman AB, Nevitt M, Stamm E (2002). Leg muscle mass and composition in relation to lower extremity performance in men and women aged 70 to 79: the health, aging and body composition study. J Am Geriatr Soc.

[34] Mcleod JC, Stokes T, Phillips SM (2019). Resistance exercise training as a primary countermeasure to age-related chronic disease. Front Physiol.

[35] Newman AB, Simonsick EM, Naydeck BL, Boudreau RM, Kritchevsky SB, Nevitt MC (2006). Association of long-distance corridor walk performance with mortality, cardiovascular disease, mobility limitation, and disability. J Am Med Assoc.

[36] Jadczak AD, Makwana N, Luscombe-Marsh N, Visvanathan R, Schultz TJ (2018). Effectiveness of exercise interventions on physical function in community-dwelling frail older people: an umbrella review of systematic reviews. JBI Database Syst Rev Implement Rep.

[37] Prevett C, Moncion K, Phillips S, Richardson J, Tang A (2022). The role of resistance training in mitigating risk for mobility disability in community-dwelling older adults: a systematic review and meta-analysis. Arch Phys Med Rehab.

[38] De Vries NM, Van Ravensberg CD, Hobbelen JS, Olde Rikkert MG, Staal JB, Nijhuis-Van Der Sanden MW (2012). Effects of physical exercise therapy on mobility, physical functioning, physical activity and quality of life in community-dwelling older adults with impaired mobility, physical disability and/or multi-morbidity: a meta-analysis. Ageing Res Rev.

[39] Lustosa LP, Silva JP, Coelho FM, Pereira DS, Parentoni AN, Pereira LS (2011). Impact of resistance exercise program on functional capacity and muscular strength of knee extensor in pre-frail community-dwelling older women: a randomized crossover trial. Rev Bras Fisioter.

[40] Thiebaud D, Jacot E, Defronzo RA, Maeder E, Jequier E, Felber JP (1982). The effect of graded doses of insulin on total glucose uptake, glucose oxidation, and glucose storage in man. Diabetes.

[41] Holloszy JO (2005). Exercise-induced increase in muscle insulin sensitivity. J Appl Physiol.

[42] Gordon BA, Benson AC, Bird SR, Fraser SF (2009). Resistance training improves metabolic health in type 2 diabetes: a systematic review. Diabetes Res Clin Pract.

[43] Yang P, Swardfager W, Fernandes D, Laredo S, Tomlinson G, Oh PI (2017). Finding the optimal volume and intensity of resistance training exercise for type 2 diabetes: the forte study, a randomized trial. Diabetes Res Clin Pract.

[44] Association AH. American heart association recommendations for physical activity in adults and kids. 2018.

[45] Kamada M, Shiroma EJ, Buring JE, Miyachi M, Lee IM (2017). Strength training and all-cause, cardiovascular disease, and cancer mortality in older women: a cohort study. J Am Heart Assoc.

[46] Macdougall JD, Tuxen D, Sale DG, Moroz JR, Sutton JR (1985). Arterial blood pressure response to heavy resistance exercise. J Appl Physiol.

[47] Hollings M, Mavros Y, Freeston J, Fiatarone SM (2017). The effect of progressive resistance training on aerobic fitness and strength in adults with coronary heart disease: a systematic review and meta-analysis of randomised controlled trials. Eur J Prev Cardiol.

[48] Srikanthan P, Horwich TB, Tseng CH (2016). Relation of muscle mass and fat mass to cardiovascular disease mortality. Am J Cardiol.

[49] Ruiz JR, Sui X, Lobelo F, Morrow JR, Jackson AW, Sjöström M (2008). Association between muscular strength and mortality in men: prospective cohort study. BMJ.

[50] Kim Y, Wijndaele K, Lee DC, Sharp SJ, Wareham N, Brage S (2017). Independent and joint associations of grip strength and adiposity with all-cause and cardiovascular disease mortality in 403,199 adults: the UK biobank study. Am J Clin Nutr.

[51] Cornelissen VA, Smart NA (2013). Exercise training for blood pressure: a systematic review and meta-analysis. J Am Heart Assoc.

[52] Lira FS, Yamashita AS, Uchida MC, Zanchi NE, Gualano B, Martins E (2010). Low and moderate, rather than high intensity strength exercise induces benefit regarding plasma lipid profile. Diabetol Metab Syndr.

[53] Sheikholeslami Vatani D, Ahmadi S, Ahmadi Dehrashid K, Gharibi F (2011). Changes in cardiovascular risk factors and inflammatory markers of young, healthy, men after six weeks of moderate or high intensity resistance training. J Sports Med Phys Fit.

[54] Grgic J, Schoenfeld BJ (2019). Higher effort, rather than higher load, for resistance exercise-induced activation of muscle fibres. J Physiol.

[55] Fisher J, Steele J, Smith D (2017). High-and low-load resistance training: interpretation and practical application of current research findings. Sports Med.

[56] Farrow J, Steele J, Behm DG, Skivington M, Fisher JP (2021). Lighter-load exercise produces greater acute-and prolonged-fatigue in exercised and non-exercised limbs. Res Q Exerc Sport.

[57] Sundstrup E, Jakobsen MD, Andersen CH, Zebis MK, Mortensen OS, Andersen LL (2012). Muscle activation strategies during strength training with heavy loading vs. repetitions to failure. J Strength Cond Res.

[58] Zourdos MC, Goldsmith JA, Helms ER, Trepeck C, Halle JL, Mendez KM (2021). Proximity to failure and total repetitions performed in a set influences accuracy of intraset repetitions in reserve-based rating of perceived exertion. J Strength Cond Res.

[59] Behm DG, Reardon G, Fitzgerald J, Drinkwater E (2002). The effect of 5, 10, and 20 repetition maximums on the recovery of voluntary and evoked contractile properties. J Strength Cond Res.

